# Off‐Label Antipsychotic Withdrawal in People With Intellectual Disabilities: Development and Internal Validation of a Prediction Model

**DOI:** 10.1111/jir.70038

**Published:** 2025-09-04

**Authors:** Joëlle Weijgertze‐Lanser, Maureen B. G. Wissing, Roy G. Elbers, Josien Jonker, Gerda M. de Kuijper, Dederieke A. M. Maes‐Festen

**Affiliations:** ^1^ Department of General Practice, Intellectual Disability Medicine Research, Erasmus MC University Medical Center Rotterdam the Netherlands; ^2^ Academic Collaborative Research Center Healthy Ageing and Intellectual Disabilities Rotterdam the Netherlands; ^3^ Department of Centre for Intellectual Disabilities and Mental Health/GGZ Drenthe the Netherlands

**Keywords:** antipsychotics, challenging behaviour, intellectual disabilities, off‐label, withdrawal

## Abstract

**Background:**

Off‐label antipsychotic use in people with intellectual disabilities and challenging behaviour is high. Antipsychotic withdrawal is recommended, but attempts are often unsuccessful. This study aimed to develop and internally validate a prediction model that provides insight into predicting factors for unsuccessful (i.e. incomplete) off‐label antipsychotic withdrawal attempts in people with intellectual disabilities.

**Methods:**

Data collected in two previous studies examining the withdrawal of off‐label antipsychotics in people with intellectual disabilities and challenging behaviour living mostly in 24/7 care settings (98.6%) in the Netherlands were analysed. The dataset included 141 participants (64.5% male, median age 52). We selected candidate predictors (age, level of intellectual disability, defined daily dose, autism spectrum disorder and three subscales of the Aberrant Behavior Checklist [ABC], namely stereotypy, hyperactivity and lethargy) based on previous research and clinical relevance. A multivariable logistic regression analysis with backward selection procedures was conducted to identify significant predictors. The model was internally validated using bootstrapping procedures.

**Results:**

The analysis revealed the level of intellectual disability (*p* = 0.030, OR = 2.374), defined daily dose (*p* = 0.063, OR = 2.833), and ABC stereotypy (*p* = 0.007, OR = 1.106) as key predictors for unsuccessful withdrawals. The variables explained 20% of the variance (Nagelkerke's *R*‐square, *R* ^2^ = 0.200). The model calibrated well as the Hosmer and Lemeshow test was not significant. The discrimination of the model was fair to good; the Area Under the Curve (AUC) was 0.728. Internal validation procedures showed an optimism‐corrected AUC of 0.706; the optimism‐corrected Nagelkerke's *R* ^2^ was 0.157.

**Conclusions:**

The odds of unsuccessful withdrawal increase with a more severe level of intellectual disability, a higher antipsychotic defined daily dose and higher stereotypy scores. The results inform healthcare providers about the predictive factors enabling them to better anticipate and support future withdrawal attempts.

## 1 Background

People with intellectual disabilities (ID) have significant limitations in intellectual and adaptive functioning (American Psychiatric Association 2013). In this population, antipsychotics are frequently prescribed. Studies indicate that 28%–33% of people with ID use antipsychotics, with more than half (58%–61%) of these prescribed off‐label (i.e., outside licenced registrations) (de Kuijper et al. 2010; García‐Domínguez et al. 2022). Off‐label antipsychotics are commonly used to treat challenging behaviour (Beumer et al. 2021), which can manifest as destructive behaviour, aggression or self‐harm (Emerson et al. 2001).

Prescribing antipsychotics to treat challenging behaviour should be considered as a temporary solution (Embregts et al. 2019). However, once prescribed, antipsychotics are frequently used for prolonged periods while challenging behaviour persists (Sheehan and Hassiotis 2017). Moreover, long‐term antipsychotic use can induce side effects, such as movement disorders and metabolic disturbances (Sheehan and Hassiotis 2017). This is particularly concerning given the lack of evidence about the effectiveness of antipsychotics as a treatment for challenging behaviour in people with ID (Tyrer et al. 2008). Therefore, there is criticism of the off‐label antipsychotic use in this population (Sheehan and Hassiotis 2017).

Reducing off‐label antipsychotic use in people with ID is a component in several measures that have been undertaken. For example, stopping overmedication of people with ID and/or autism (STOMP) in the United Kingdom (Shankar et al. 2019), the Dutch Guideline for Challenging (Embregts et al. 2019) and the Dutch Care and Coercion Act (*Wet zorg en dwang psychogeriatrische en verstandelijk gehandicapte clienten* 2020). Despite the implementation of these measures, off‐label antipsychotic withdrawal in people with ID is often unsuccessful (Beumer et al. 2021).

Previous studies have reported several factors that may influence the outcome of off‐label antipsychotic withdrawal attempts in people with ID. Several of these factors, such as a more severe level of intellectual disability (de Kuijper and Hoekstra 2018; Stevenson et al. 2004), the presence of autism spectrum disorder (ASD) and akathisia (antipsychotic induced movement disorder) (de Kuijper and Hoekstra 2018) were associated with unsuccessful—that is, incomplete withdrawal attempts. Additionally, previous research has shown that the attitude of caregivers towards a withdrawal attempt is related to the withdrawal outcome (de Kuijper et al. 2022; Ahmed et al. 2000). Furthermore, Sheehan and Hassiotis (2017) reported in their systematic review that unsuccessful withdrawals were associated with higher baseline antipsychotic dose, earlier unsuccessful withdrawal attempts, comorbid psychiatric disorders and not using other psychotropic drugs (Sheehan and Hassiotis 2017). Although several factors have been linked to unsuccessful withdrawal attempts, contradictory results regarding the outcome of off‐label antipsychotic withdrawal attempts have been found for sex (de Kuijper et al. 2014; Hancock et al. 1991) and the severity of behavioural problems (de Kuijper et al. 2014; Janowsky et al. 2008; Branford 1996; May et al. 1995).

Previous research in people with ID has predominantly focused on associations between off‐label antipsychotic withdrawal outcomes and patient characteristics. However, findings have been contradictory, highlighting the need for further research on withdrawing antipsychotics in people with ID. A prediction model may serve as a structured approach to identify potentially relevant predictors, thereby laying the groundwork for future research and more actionable tools. This would enable clinicians to anticipate future withdrawal attempts. To the best of our knowledge, a prediction model has not yet been developed. Therefore, this study aims to develop and internally validate a prediction model that provides insight into predicting factors for unsuccessful off‐label antipsychotic withdrawal attempts in people with ID.

## 2 Methods

### 2.1 Study Design

We combined and analysed baseline and withdrawal outcome data of two antipsychotic withdrawal studies in people with ID (Beumer et al. 2021; de Kuijper et al. 2014). We examined which baseline factors could predict the outcome of an off‐label antipsychotic withdrawal attempt. We reported the results in accordance with the TRIPOD‐Statement (Collins et al. 2024).

### 2.2 Data Sources

#### 2.2.1 Study 1

Study 1 was an open‐label parallel‐group withdrawal trial (EudraCT 2007‐005451‐42‐NL) (de Kuijper et al. 2014). Participants were recruited from three care organisations for people with ID in the Netherlands. In total, 98 participants with ID, aged 15–66 years using antipsychotics (risperidone, pipamperone, haloperidol, levomepromazine, pimozide and/or olanzapine) off‐label for at least 1 year, were included. Participants were randomly allocated into two groups with a withdrawal schedule of either 14 or 28 weeks to compare the results of two withdrawal schedules (1:1 ratio). Antipsychotics were withdrawn in eight intended dose decreases of approximately 12.5% of the initial dose, every 2 or 4 weeks, respectively. The withdrawal was stopped if the participant, caregivers and/or clinicians found it necessary to stop, for example, if the participant experienced behavioural worsening. Data were extracted from medical records and questionnaires completed by caregivers. Study 1 was conducted between 2009 and 2011.

The Medical Ethical Committee of the University Medical Centre Groningen (MEC 2008.232) approved Study 1. Participants or their legal representatives gave consent for follow‐up research. For a more detailed overview of Study 1, see de Kuijper et al. (2014).

#### 2.2.2 Study 2

Study 2 was a double‐blind placebo‐controlled withdrawal trial (EudraCT 2016‐002859‐19‐NL) (Beumer et al. 2021). Participants were recruited from five care organisations for people with ID in the Netherlands. In total, 87 participants with ID, aged 18 years or older using off‐label antipsychotics (risperidone or pipamperone) for at least 1 year, were included. Participants were allocated into a withdrawal and a control group (1:1 ratio). Antipsychotics were replaced by placebo in 25% dose reductions every 4 weeks, followed by a 6‐week double‐blind period. Blinding was broken after 22 weeks. If the participant, caregivers and/or clinicians found it necessary to stop the withdrawal for example, if there was deterioration in behaviour, blinding was broken earlier. Data were extracted from questionnaires completed by caregivers and physical examinations. Study 2 was conducted between 2019 and 2021. For the study described in this article, we only included data from the withdrawal group (*n* = 43).

The Medical Ethical Committee of the Erasmus University Medical Centre Rotterdam (MEC‐2016‐573) approved Study 2. Participants or their legal representatives gave consent for follow‐up research. For a more detailed overview of Study 2, see the protocol article of Beumer et al. (2021).

### 2.3 (Un)successful Withdrawal

Data from both studies on the outcome of withdrawal at the end of the intervention period were used as the outcome measure for the development of the prediction model. The outcome was dichotomous, meaning that the withdrawal was either successful when the participant fully withdrew to zero at the end of the intervention period (completion) or unsuccessful when discontinued prematurely. This approach does not account for partial withdrawals, such as withdrawing to 25% of the original dose, which in clinical practice may still be considered successful.

### 2.4 Candidate Predictors

In three consensus meetings, relevant baseline variables to include in the prediction model were discussed and selected based on availability in the two datasets, clinical expertise and literature (Sheehan and Hassiotis 2017; de Kuijper and Hoekstra 2018; Stevenson et al. 2004; de Kuijper et al. 2014; Hancock et al. 1991; Janowsky et al. 2008; Branford 1996; May et al. 1995). Members of the consensus group were two ID physicians, a human movement scientist, a registered nurse and a behavioural scientist, all specialised in ID care. The consensus team included professionals with practical experience in the care and support of people with ID, as well as professionals with a broad academic background. This ensured that the perspective of the field and the scientific perspective were well represented throughout the selection process of predictors. Candidate predictors identified in the first consensus meeting were age, sex, living situation, level of intellectual disability, presence of sensory impairments, comorbid psychiatric disorders, ASD, physical multimorbidity, dyskinesia (a movement disorder), sedative medication use, defined daily dose (DDD) and subscale scores of Aberrant Behavior Checklist (ABC) (Aman and Singh 1991). The ABC is a questionnaire to evaluate the effect of treatment on challenging behaviour in people with ID, consisting of five subscales: irritability, lethargy, stereotypy, hyperactivity and inadequate speech. Items were scored on a 4‐point scale to assess the severity of problems, with high scores indicating more severe problems (Aman and Singh 1991).

### 2.5 Preparation of Candidate Predictors

To facilitate merging the two datasets, variables were recoded into categories. The presence of sensory impairments was scored positive if the participants' medical file indicated blindness, visual impairments, deafness or hearing impairments. Comorbid psychiatric disorder was scored present if the participant had one or more of the following disorders documented in their medical or behavioural file: anxiety disorders, mood disorders, obsessive compulsive disorder, attachment disorder, attention deficit hyperactivity disorder and other psychiatric disorders. ASD was included separately in the dataset because previous research showed an association between ASD and withdrawal outcome (de Kuijper and Hoekstra 2018). ASD was scored present if a clinical diagnosis was reported in the medical or behavioural file of the participant. We defined physical multimorbidity as the presence of two or more chronic physical disorders reported in the medical file. An overview of physical disorders encompassed by this variable can be found in Table S1. Dyskinesia was measured with the Abnormal Involuntary Movement Scale (AIMS) (Guy 1976) in Study 1 and with the St Hans Rating Scale (SHRS) (Gerlach et al. 1993) in Study 2. We recoded the scores of the scales to ensure comparability. Dyskinesia was scored present if there was at least one moderate or two or more mild scores on the scale items, which is in accordance with the criteria of Schooler and Kane (1982). The variable sedative medication use comprised medication belonging to the WHO ATC N05 (excluding antipsychotics) classification (WHO 2024). Sedative medication was scored as present if one or more N05 classified medication was recorded on the participant's drug list. As needed, medication was not included in this variable. DDD was calculated according to the WHO ATC/DDD Index 2024. For each antipsychotic, the total prescribed dose was divided by the corresponding DDD (WHO 2024).

### 2.6 Selection of Candidate Predictors

To prevent the Events Per Variable Problem (EVP), wherein regression coefficients may be overestimated if too many variables are included, we adhered to the rule of thumb of 10–15 events per variable (degree of freedom) (Harrell et al. 1985; Peduzzi et al. 1995, 1996). Examination of the frequency table of the withdrawal outcome indicated the potential inclusion of seven predictors in the prediction model.

**FIGURE 1:**
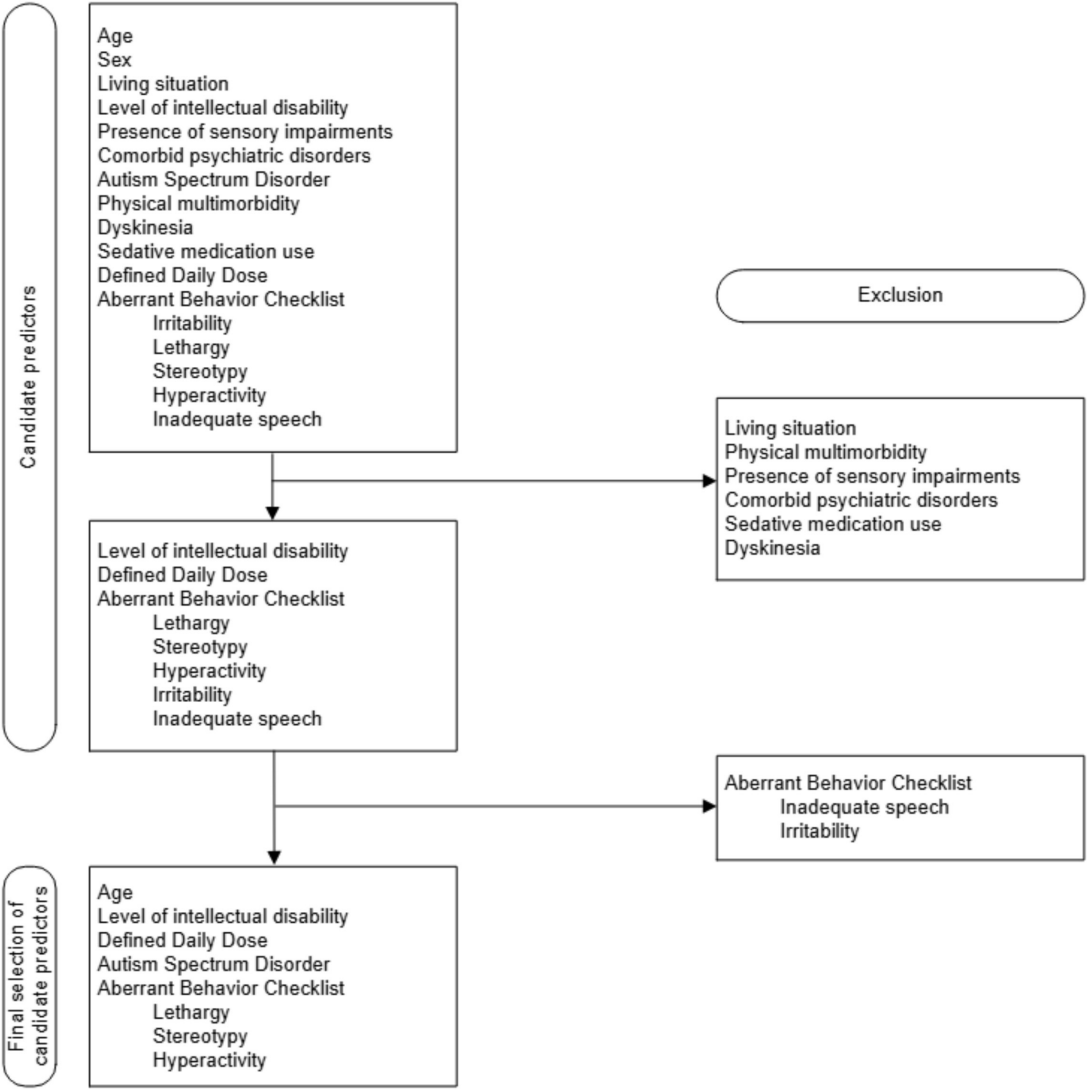
Flowchart of selection of candidate predictors for the prediction model.

Because more than seven candidate predictors were identified in the first consensus meeting, a second consensus meeting was held to refine the variable selection. A visual overview of the selection of candidate predictors is shown in Figure 1. Candidate predictors were not included due to three reasons. First, almost all participants lived in a 24/7 residential setting; therefore, we decided not to include living situation. Second, the variable physical multimorbidity consists of varying disorders, which makes it less reliable to include this variable as a predictor. Third, other variables seemed more important for the outcome according to clinical expertise than the presence of comorbid psychiatric disorders, sensory impairments, sedative medication use and dyskinesia. We did not reach consensus regarding the ABC subscales. Therefore, univariable regression analyses were performed (*α* = 0.1) to determine which ABC subscales had the largest association with unsuccessful withdrawal. Results of the univariable analyses were discussed in the third consensus meeting. As a result, ABC lethargy (*p* = 0.015), ABC stereotypy (*p* < 0.001) and ABC hyperactivity (*p* = 0.011) were selected. ABC irritability was also significantly (*p* = 0.016) associated with unsuccessful withdrawal but not included due to high correlations with ABC hyperactivity. To reduce the degrees of freedom, the level of intellectual disability was categorised into two levels: mild and moderate ID and severe and profound ID. This resulted in the final selection of the following baseline candidate predictors: age, level of intellectual disability, DDD, ASD, ABC lethargy, ABC stereotypy and ABC hyperactivity.

### 2.7 Data Analysis

#### 2.7.1 Baseline Characteristics and Withdrawal Outcome

Descriptive statistics were used to present baseline characteristics. To examine differences in baseline characteristics between the successful and unsuccessful withdrawal groups, Mann–Whitney *U* tests were carried out for nonnormally distributed continuous variables. For categorical variables, chi‐squared tests were carried out. To examine differences in the withdrawal outcome between the two studies, a chi‐squared test was performed.

#### 2.7.2 Model Development and Evaluation

We performed a multivariable logistic regression analysis with the final selection of candidate predictors (age, level of intellectual disability, DDD, ASD, ABC lethargy, ABC stereotypy and ABC hyperactivity) to examine if variables were predictive of unsuccessful withdrawal attempts. We used a backward selection method (*α* = 0.10). Participants with missing data on the selected candidate predictors were excluded from the analysis. This did not affect the number of variables that we could include in the prediction model according to the EVP. To control for significant differences in withdrawal outcomes between the two studies, data source (Study 1 or 2) was included as a covariate in the model development analysis. We did not include the duration of withdrawal as a covariate because de Kuijper et al. (2014) did not find a significant difference in withdrawal schedule and withdrawal outcome.

To ensure the quality of the model, the Nagelkerke's *R*‐square (*R* ^2^) was examined and interpreted as the proportion of explained variance (Nagelkerke 1991). We evaluated the calibration of the model using the Hosmer–Lemeshow goodness of fit and graphically with a calibration plot. Calibration is the agreement between the predicted probabilities and the observed outcomes (Steyerberg et al. 2010). A nonsignificant *p* value for the Hosmer–Lemeshow test was sought. Finally, the discriminative ability of the model was evaluated using the Receiver Operating Characteristic (ROC) curve and the Area under ROC (AUC). The AUC gives an indication of how well the model distinguishes between those with and without the outcome (Steyerberg et al. 2010).

After development, the model was internally validated using bootstrapping procedures. We drew 1000 bootstrap samples to assess the degree of optimism of the prediction model. A shrinkage factor was applied to correct the model for optimism. Baseline characteristics and differences in withdrawal outcome were analysed using SPSS Statistics (IBM, Version 28). R Version 4.4.2, package ‘rms’ (Harrell, n.d.), was used to develop and internally validate the prediction model.

## 3 Results

Seventy participants (49.9%) of the total 141 participants did not successfully withdraw from the antipsychotic(s) at the end of the intervention period. A chi‐squared test revealed that withdrawal was more often unsuccessful in participants in Study 1 (*n* = 98; 56.1%) compared to participants in Study 2 (*n* = 43; 34.9%; *p* = 0.020).

Baseline characteristics of 141 participants are shown in Table 1. Significant differences were found between the successful and unsuccessful withdrawal groups (Table 1). The unsuccessful withdrawal group contained more males (*p* = 0.016) and more participants with a severe/profound level of intellectual disability (*p* = 0.013). Moreover, more participants used haloperidol (*p* = 0.040) and levomepromazine (*p* = 0.050) in the unsuccessful withdrawal group. Additionally, sedative medication was more frequently used in the unsuccessful withdrawal group (*p* = 0.036). The DDD was found to be higher in the unsuccessful withdrawal group (*p* = 0.002). Finally, higher mean ABC subscale scores were found in the unsuccessful withdrawal group for subscales ABC irritability (*p* = 0.019), ABC lethargy (*p* = 0.008), ABC stereotypy (*p* ≤ 0.001) and ABC hyperactivity (*p* = 0.018).

**TABLE 1:** Baseline characteristics.

	Total (*N* = 141)	Unsuccessful (*n* = 70)	Successful (*n* = 71)	*p* value^a^
Sex, *n* (% male)	91 (64.5)	52 (74.3)	39 (54.9)	0.016*
Age, median (IQR) [range]	52.00 (22.00) [20–81]	50.00 (23.00) [20–81]	54.00 (21.00) [21–78]	0.606
Level of ID, *n* (%)				0.013*
Mild/moderate	59 (41.8)	22 (31.4)	37 (52.1)	
Severe/profound	82 (58.2)	48 (68.6)	34 (47.9)	
Living situation, *n* (%)				0.369
24/7 care	139 (98.6)	70 (100)	69 (97.2)	
Partial	1 (0.7)	0 (0)	1 (1.4)	
Ambulatory	1 (0.7)	0 (0)	1 (1.4)	
Type of antipsychotic, *n* (%)				
Pimozide	1 (0.7)	0 (0)	1 (1.4)	0.319
Haloperidol	18 (12.8)	13 (18.6)	5 (7.0)	0.040*
Levomepromazine	7 (5.0)	6 (8.6)	1 (1.4)	0.050*
Olanzapine	8 (5.7)	6 (8.6)	2 (2.8)	0.140
Risperidone	29 (20.6)	14 (20.0)	15 (21.1)	0.869
Pipamperone	93 (66.0)	43 (61.4)	50 (70.4)	0.260
Comorbid psychiatric disorders, *n* (%)	70 (49.6)	39 (55.7)	31 (43.7)	0.152
ASD, *n* (%)	61 (43.6)	31 (44.9)	30 (42.3)	0.750
Sedative medication use, *n* (%)	41 (29.1)	26 (37.1)	15 (21.1)	0.036*
DDD, median (IQR) [range]	0.40 (0.42) [0.05–2.20]	0.50 (0.55) [0.10–1.80]	0.30 (0.45) [0.05–2.20]	0.002**
Sensory impairments, *n* (%)	56 (39.7)	24 (34.3)	32 (45.1)	0.191
Physical multimorbidity, *n* (%)	73 (51.8)	33 (47.1)	40 (56.3)	0.275
ABC, median (IQR) [range]				
Irritability	12.00 (18.00) [0–40.00]	15.00 (21.00) [0–40.00]	9.00 (16.00) [0–34.00]	0.019*
Lethargy	10.00 (14.00) [0–42.00]	11.50 (26.00) [0–42.00]	7.00 (13.25) [0–42.00]	0.008**
Stereotypy	3.00 (8.00) [0–21.00]	6.00 (10.00) [0–21.00]	1.50 (5.00) [0–18.00]	< 0.001**
Hyperactivity	10.00 (13.00) [0–36.00]	11.50 (16.50) [0–36.00]	9.00 (12.00) [0–30.00]	0.018*
Inadequate speech	1.00 (5.00) [0–12.00]	1.00 (5.00) [0–12.00]	1.00 (5.00) [0–11.00]	0.287

*Note:* Results were rounded in accordance with standard rounding conventions.

Abbreviations: ABC, Aberrant Behavior Checklist; ASD, autism spectrum disorder; DDD, defined daily dose; ID, intellectual disability.

^a^ Mann–Whitney U tests for continuous variables; chi‐squared tests for categorical variables.

* Significant at *p* ≤ 0.05.

** Significant at *p* ≤ 0.01.

### 3.1 Model Development

A logistic regression analysis using backward selection procedures on the seven candidate predictors revealed that the level of intellectual disability (*p* = 0.030, OR = 2.374 [1.086–5.191]), DDD (*p* = 0.063, OR = 2.833 [0.945–8.497]) and ABC stereotypy (*p* = 0.007, OR = 1.106 [1.028–1.190]) were predictors for the outcome of off‐label antipsychotic withdrawal (Table 2). Results showed that for a severe/profound intellectual disability, the odds of unsuccessful withdrawal increased by 137.4%. For each increase of 0.01 in DDD, the odds of unsuccessful withdrawal increased by 1.1%. Finally, for each increase in ABC stereotypy score, the odds of unsuccessful withdrawal increased by 10.6%. The analysis was repeated with the inclusion of data source (Study 1 or 2) as a covariate. The results were consistent with those obtained without including the covariate. This showed that the source of data did not significantly influence the outcome of the model.

**TABLE 2:** Final prediction model.

	Exp(*B*)	SE	*p* value	OR (95% CI)
Intercept	−1.558	0.438	< 0.001	0.205
Level of ID	0.865	0.399	0.030*	2.374 (1.086–5.191)
DDD	1.041	0.560	0.063	2.833 (0.945–8.497)
ABC stereotypy	0.101	0.037	0.007**	1.106 (1.028–1.190)

*Note: N* = 136.

Abbreviations: ABC, Aberrant Behavior Checklist; ASD, autism spectrum disorder; CI, confidence interval; DDD, defined daily dose; Exp(*B*), exponentiated coefficient; ID, intellectual disability; OR, odds ratio; SE, standard error.

* Significant at *p* ≤ 0.05.

** Significant at *p* ≤ 0.01.

### 3.2 Model Performance

**FIGURE 2:**
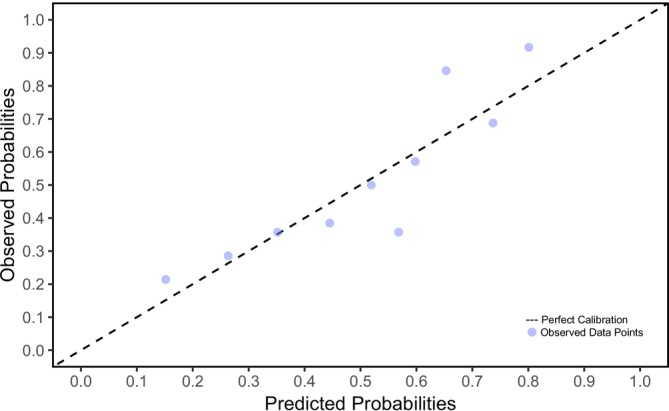
Calibration plot of the prediction model. The points to the diagonal line indicate the model's accuracy.

**FIGURE 3:**
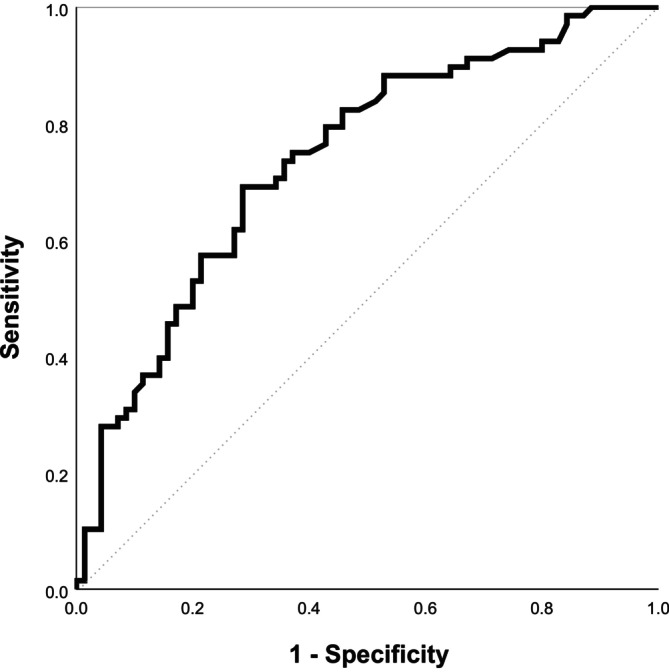
ROC curve of the prediction model. The true positive rate (sensitivity) is plotted against the false positive rate (1‐specificity).

The prediction model explained 20% of the variance (Nagelkerke's *R* ^2^ = 0.200) and had a moderate fit to the data. Calibration was assessed using the Hosmer and Lemeshow test. The Hosmer and Lemeshow test was not significant (*p* = 0.776), which indicated that the model calibrated well. The calibration plot is shown in Figure 2. The AUC was 0.728, indicating that the discrimination of the prediction model was fair to good. To illustrate the discriminative ability of the model, the ROC curve is shown in Figure 3. Internal validation procedures with bootstrapping (1000 samples) showed an optimism corrected AUC of 0.706; the optimism corrected Nagelkerke's *R* ^2^ was 0.157.

## 4 Discussion

In this study, we aimed to develop a prediction model for off‐label antipsychotic withdrawal in people with ID. We found that the odds for unsuccessful withdrawal increase with a more severe level of intellectual disability, higher DDD and higher ABC stereotypy scores. The predictors accounted for 20% of the variance. The model calibrated well, and the discrimination of the model was fair to good after internal validation procedures.

There were differences in the withdrawal outcome in the two datasets that were used to develop the prediction model. Withdrawal was significantly more often unsuccessful in participants in Study 1. A possible explanation might be that participants in Study 1 were aware that they withdrew from the antipsychotic(s). A fear of behavioural worsening during antipsychotic withdrawal might have influenced the perspective of the caregivers on the behaviour of the participant negatively (de Kuijper et al. 2022). In Study 2, neither participants nor caregivers were aware of the antipsychotic withdrawal status, potentially leading to less unsuccessful withdrawal compared to Study 1. Another explanation may be that participants in Study 2 were more motivated to complete the double‐blind period to avoid deblinding based on the assumption that they were in the withdrawal group, because of the possibility that they were in the control group and thus having been incorrect in their assumptions about withdrawing the antipsychotics. Although there was a significant difference in the withdrawal outcome of Studies 1 and 2, including data source as a covariate did not affect the results of the prediction model. This indicates that the findings were not influenced by the differences in the withdrawal outcome.

Based on clinical expertise and previous research, it was expected that the variables age, level of intellectual disability (de Kuijper and Hoekstra 2018; Stevenson et al. 2004), DDD (de Kuijper and Hoekstra 2018; Branford 1996; May et al. 1995; Deb et al. 2020), ASD (de Kuijper and Hoekstra 2018) and the three subscales of the ABC (de Kuijper et al. 2014; Janowsky et al. 2008; Branford 1996; May et al. 1995) were predictive of the outcome of off‐label antipsychotic withdrawals. However, not every variable emerged as a significant predictor in this study. First, age was included in the analysis. We hypothesised that withdrawal of antipsychotics would be more feasible in older patients with ID as challenging behaviour tends to occur more frequently in young adults (Holden and Gitlesen 2006). However, our analysis did not confirm this hypothesis. An explanation for this may be that age does not directly influence the withdrawal outcome but rather reflects other underlying severe and chronic problems that are more prevalent among older individuals (WHO 2016), yet were not included in the model. Second, ASD was included in our analysis because ASD was found to be associated with unsuccessful antipsychotic withdrawals in people with ID (de Kuijper and Hoekstra 2018). However, ASD did not emerge as a significant predictor of the withdrawal outcome in our study. This difference may be related to the difficulty of diagnosing ASD in people with ID (Matson and Shoemaker 2009). In our study, ASD was only considered present if a clinical diagnosis of ASD was reported in the participant's medical or behavioural file. Participants were not screened for ASD, and differential diagnoses were not considered.

Three variables emerged as predictors for the withdrawal outcome. First, the level of intellectual disability was found predictive for the outcome of an off‐label antipsychotic withdrawal attempt, in alignment with the findings of previous studies (de Kuijper and Hoekstra 2018; Stevenson et al. 2004). An explanation for the predictive value of the level of ID may be that people with severe/profound ID may suffer from severe behavioural problems due to their limited communication skills, which may have been the indication of the antipsychotic prescription and which might hinder a successful withdrawal (Bowring et al. 2017). Additionally, DDD was found predictive for the withdrawal outcome. Other studies also found associations between the extent of baseline dosage (DDD) and the withdrawal outcome (de Kuijper and Hoekstra 2018; Branford 1996; May et al. 1995; Deb et al. 2020). One explanation for this might be that for people with a higher DDD, the withdrawal steps (12.5% and 25.0% of the original dose) represent a larger absolute decrease of the dosage, whereas for people with a lower DDD, these steps correspond to a smaller absolute change in dosage. This may result in lower withdrawal success rates for people with a higher DDD. Furthermore, higher ABC stereotypy scores were predictive for unsuccessful withdrawal in our study. The associations between ABC scores and the withdrawal outcome were also examined by Branford (1996). He found that lower ABC stereotypy scores were associated with successful withdrawals, compliant with the findings of our study. The exact mechanism explaining why stereotypy is predictive for the withdrawal outcome remains difficult to unravel. As ASD did not emerge as a significant predictor, we did not find an association between ASD and stereotypy. Further research is needed to gain a deeper understanding of this phenomenon.

To the best of our knowledge, this is the first study that aimed to develop a prediction model for the outcome of off‐label antipsychotic withdrawal attempts in people with ID. It is unique to combine data from two independent antipsychotic withdrawal studies, as these kinds of intervention studies are limited in the population of people with ID. A limitation of the study is that merging data from two independent studies was challenging. For example, de Kuijper and Hoekstra (2018) found associations with parkinsonism and akathisia and withdrawal outcome. However, it was impossible to combine these variables from both studies because parkinsonism and akathisia were measured in different ways. In addition, due to the 10‐year gap between the two studies, Study 2 included relevant variables in the dataset according to recent literature, whereas Study 1 had not included these variables. Consequently, variables such as previous withdrawal attempts (Sheehan and Hassiotis 2017) and contextual factors such as the attitude of caregivers towards withdrawal (de Kuijper et al. 2022; Ahmed et al. 2000) were deemed important in the literature but could not be included in our analysis. A second limitation of the study is that although we merged two datasets, the sample size is still relatively small. Due to the small sample size, we could only include seven variables and therefore needed to exclude potentially relevant variables such as sex (de Kuijper et al. 2014; Hancock et al. 1991). The inclusion of seven variables was based on the statistical rule of 10–15 events per variable (Harrell et al. 1985; Peduzzi et al. 1995; Peduzzi et al. 1996). However, it is suggested that 10–15 events per variable is overrated (Vittinghoff and McCulloch 2006). Moreover, because we merged the two datasets, it was not possible to externally validate the prediction model as there was no other dataset available. A final limitation is that this study did not include follow‐up data but only assessed the withdrawal outcome at the end of the intervention period. We did not include these data in the analysis because both studies had different follow‐up durations.

In this study, candidate predictors were primarily nonmodifiable factors. In future research, contextual factors should also be considered, so that clinical practice is not only informed by the predictors but can also further improve contextual factors before starting a withdrawal. Additionally, an accessible international database containing anonymised data from intervention studies or healthcare data on antipsychotic withdrawal in people with ID would enable replication of our study with inclusion of more variables. It would also become feasible to apply machine learning techniques to improve the predictive accuracy of the model. Moreover, it would be possible to externally validate the prediction model. Based on the results of the externally validated prediction model, a prediction tool can be developed to guide practice. That is beyond the scope of this study.

Despite the limitations of this study, we could explain 20% of the variance. The results of the study can contribute to raising awareness among healthcare providers, carers and people with ID about the factors that are predictive for the outcome of an off‐label antipsychotic withdrawal attempt. Healthcare providers can anticipate and support future antipsychotic withdrawals by providing targeted support to the caregivers or taking preventive measures, such as adopting a different, slower withdrawal schedule when predictive factors are present. This may contribute to improving the probability of a successful withdrawal. It is important to note that this study employed a dichotomous outcome measure (successful or unsuccessful withdrawal). This does not always justify the complexity of practice. Some cases that were classified as unsuccessful in this study may be (partly) perceived as successful withdrawal in clinical practice. This is further illustrated by the finding that 80% of the variance was not explained by the variables in the final prediction model. This means that withdrawal might be influenced by random factors that are too complex to systematically explain or factors that were not measured in this study. This supports the understanding that off‐label antipsychotic withdrawal is a complex process, which still needs to be better understood. Although the findings provide insight into the predicting factors for off‐label antipsychotic withdrawal, it is important to acknowledge a broader dilemma in clinical practice. The decision whether or not to withdraw can be a very complex decision where practitioners are faced with resistance and fear of withdrawal. They encounter the potential risk of symptom worsening and disruption, despite lack of evidence for the use of off‐label antipsychotics, side effects and guidelines for withdrawal.

To conclude, we found that a more severe level of intellectual disability, a higher baseline DDD and higher ABC stereotypy scores increased the odds of unsuccessful off‐label antipsychotic withdrawal in people with ID. This study laid the groundwork for future research and more actionable tools by identifying relevant predictors for off‐label antipsychotic withdrawal attempts in people with ID. The study increased insight into predicting factors, which allows healthcare providers to anticipate future withdrawal attempts.

## Ethics Statement

The Medical Ethical Committee of the Erasmus University Medical Centre Rotterdam approved the study reported in this article (MEC‐2023‐0690).

## Conflicts of Interest

The authors declare no conflicts of interest.

## Supporting information


**Table S1:** Overview of included chronic disorders in the variable ‘physical multimorbidity’.

## Data Availability

Individual participant data will not be available, as this article presents secondary data analyses.
