# Supplementary material for: Off‐Label Antipsychotic Withdrawal in People With Intellectual Disabilities: Development and Internal Validation of a Prediction Model
Source: J Intellect Disabil Res. 2025 Sep 4;69(10):1042–50. doi: 10.1111/jir.70038 (PMC12576382; doi:10.1111/jir.70038)
Supplement: Supplementary file 1 — Table S1: Overview of included chronic disorders in the variable ‘physical multimorbidity’. [file JIR-69-1042-s001.pdf]

Supplementary material

Table of Contents

List of the encompassed chronic disorders in the variable physical multimorbidity 2

List of the encompassed chronic disorders in the variable physical multimorbidity

Physical multimorbidity was scored present if the participant had two or more chronic physical disorders (in the column ‘category’) in their medical file.

Supplementary Table 1. Overview of included chronic disorders in the variable 'physical multimorbidity'

**Category**	**Including**
Internal disease	Hepatitis
	Gallbladder
Gastrointestinal	Stomach problems
	Reflux
	Rumination
	Gastrointestinal disorder
	Obstipation
	Irritable bowel
	Inflammatory bowel diseases
	Dental problems
	Swallowing disorders
Kidneys/urinary tract	Incontinence
	Urinary retention
	Urinary stone disease
	Kidney failure
	Penile disorders
	Prostate diseases
	Venereal diseases
	Hypogonadism
Musculoskeletal	Joint problems
	Scoliosis
	Osteoporosis
	Contractures
Neurological conditions	Migraine
	Spinal stenosis
	Carpal tunnel syndrome
	Hernia
	Paralysis
	Cerebral parese
	Hypotonia
	Spasticity
	Parkinson’s disease
	Sleeping problems
Endocrine disorders	Menstrual complaints
	Menopause symptoms
	Hypothyroid
	Hyperthyroid
	Hyperprolactinemia
	Other endocrine conditions

	Diabetes Mellitus1
	Diabetes Mellitus 2
	Cushing’s syndrome
Skin conditions	Decubitus
	Skin conditions
Surgical	Inguinal hernia
Cardiovascular diseases	Heart disease
Lung diseases	Asthma/copd
Metabolic disorders	Metabolic syndrome
Haematological	Anaemia
Tumours	Cancer
Allergy	Allergy
Celiac	Celiac
